# Defining psychological capacity for a measure of healthy ageing: a rapid scoping review

**DOI:** 10.1007/s10433-026-00933-w

**Published:** 2026-07-11

**Authors:** Candice Oster, Alice Windle, Sonia Hines, Neta Hagani, Jyoti Khadka, Vivian Isaac

**Affiliations:** 1 College of Health and Enablement, Caring Futures Institute, Flinders University, South Australia, Sturt Rd, Bedford Park, SA Australia; 2 JBI, School of Public Health, Adelaide University, Adelaide, SA Australia; 3 School of Allied Health, Exercise and Sports Sciences, Faculty of Science, Charles Sturt University, PO Box 789, Albury, NSW 2640 Australia

**Keywords:** Psychological capacity, Healthy ageing, Older people

## Abstract

Psychological capacity is a key element of intrinsic capacity for healthy ageing; however, it has not been well defined, and no established measure exists. Existing studies predominantly use a deficit framing when measuring psychological capacity, which does not align with the World Health Organization’s intrinsic capacity framework of healthy ageing. The objective of this rapid scoping review was to identify definitions and attributes of positive psychological capacity to inform development of a measure of psychological capacity for healthy ageing. This review included English-language studies of any design that provided either a specific conceptual or operational definition of psychological capacity or psychological capital, or named attributes as being related to psychological capacity or capital. Searches were conducted in PsycINFO, PubMed, Embase and Scopus for studies published between 1 January 2010 and 16 April 2025. Retrieved citations were screened and assessed against the inclusion criteria by two independent reviewers. Text data relating to definitions and descriptions of psychological capacity and psychological capital were extracted and collated. Psychological capacity attributes were inductively identified and grouped into categories. Of the 603 studies that were screened, 78 were included. Only two (2.5%) of the included studies related to older adults. The majority of studies (*n* = 46, 59%) related to the context of ‘work’. Eight different definitions of psychological capacity were identified, as well as several descriptions. Fifty-six attributes of psychological capacity were identified in included studies, and these were grouped into nine domains. There was considerable heterogeneity in definitions of psychological capacity, and no definition specific to healthy ageing. The vast majority of the literature related to the workplace context, driven by goals of performance and competitive advantage, rather than healthy ageing. Our study highlights the need for a strength-based definition and identification of attributes of psychological capacity for healthy ageing.

**Supplementary Information:**

The online version contains supplementary material available at https://doi.org/10.1007/s10433-026-00933-w.

## Introduction

Thanks to advances in medicine and public health, the global population is rapidly ageing (World Health Organization 2023), and while this represents significant success for humankind, greater longevity does not always come with good health. As such, population ageing has major implications for public health and health care systems worldwide. This has led to a global emphasis on healthy ageing to enhance quality of life for older people and mitigate demand for care and services associated with age-related needs (World Health Organization 2020).

In 2015, the World Health Organization (WHO) released a public health framework for healthy ageing (World Health Organization 2015) and proposed ‘intrinsic capacity’ as a key determinant. Intrinsic capacity refers to all the physical and mental capacities that an individual can draw on at any point in time, including locomotor capacity, cognitive capacity, sensory capacity, vitality, and psychological capacity (World Health Organization 2015). Intrinsic capacity sits alongside functional ability and environments as a component of healthy ageing (World Health Organization 2020). Monitoring countries’ progress towards healthy ageing is a key aim of WHO’s Decade of Healthy Ageing, which includes measuring intrinsic capacity and its component capacities (World Health Organization 2020).

To date, psychological capacity as a component of intrinsic capacity for healthy ageing is poorly understood (Oster et al. 2023a). The ageing literature has discussed several positive attributes such as resilience, sense of coherence, autonomy, resourcefulness, identity, hope, religiosity/spirituality, life valuation, self-efficacy, community and well-being (Stoner et al. 2018; Stoner 2019). However, there is considerable heterogeneity in how psychological capacity is defined and measured (George et al. 2021) and a tendency to use absence of depression as a proxy for psychological capacity in older people. This emphasizes deficit rather than skills, strengths or capacity for healthy ageing.

Deficit-based approaches in psychology have long been critiqued for an emphasis on individual impairments. Deficit-based framing of psychological capacity is problematic in that it: (a) assumes that mental health and mental illness are opposites along a continuum of possible states (Zhao and Tay 2023); (b) has negative effects on consumers and health professionals (Oster et al. 2023b); and (c) limits opportunities for developing interventions to support psychological capacity (Iasiello et al. 2024) as we age. By contrast, intrinsic capacity—focussing on functional ability—is grounded on capability or capacity approaches, conceptualizing well-being in terms of capabilities made of individual’s bodily functioning, knowledge and skills combined with their external social and physical conditions (Venkatapuram and Amuthavalli Thiyagarajan 2023).

Over the past two decades, there has been growing emphasis on recognizing and building people’s strengths and capacities, with the parallel evolution of positive psychology and strength-based perspectives in mental health care (Caiels et al. 2023; Seligman and Csikszentmihalyi 2014; Rapp and Goscha 2011; Rapp and Chamberlain 1985; Rapp 1998). Many fields of research including education (Chodkiewicz and Boyle 2017), health (Park et al. 2016), workplace (Donaldson et al. 2019) and sports (Gabana 2019) have adopted positive psychology and strength-based perspectives to better capture individual capacities that can be measured and developed to improve health, well-being and productivity.

Positive psychology is an umbrella term for the study of individuals’ positive attributes, psychological assets and strengths (Seligman et al. 2005; Kobau et al. 2011). Similarly, a strength-based perspective focuses on individual capacities, knowledge, abilities and other positive qualities to solve problems and promote well-being. These frameworks have contributed to a growing body of evidence on a broad set of positive attributes and experiences and the processes that contribute to improved outcomes. While several positive attributes, traits and experiences have been studied as critical indicators of mental health and well-being in older adults, there is limited understanding on the significance of the higher-order construct of psychological capacity (also known as ‘psychological capital’ and abbreviated to PsyCap) (Luthans et al. 2004). A new approach to understanding and measuring psychological capacity in older people is needed, which shifts the emphasis from deficit to functioning.

Positive psychological attributes can be classified by their function in ageing and adaptation. Some serve as antecedents or assets, relatively stable, trait-like characteristics such as self-efficacy or optimism that provide a foundation for adaptive functioning. Others represent processes or state-like capacities, such as coping or emotional regulation, that enable individuals to draw on these assets in daily life. Finally, some reflect experiential outcomes, including well-being or life satisfaction, which result from the effective use of psychological capacities. Clarifying these distinctions is essential for developing measures that accurately capture psychological capacity as a core resource for healthy ageing.

Our recent systematic review found that there is no consensus on a conceptual definition nor a single measure encompassing the broad concept of psychological capacity in older adults (Oster et al. 2023a). Prior to developing a measure of psychological capacity in older adults, we need to clearly define what is meant by psychological capacity (a conceptual definition) and how it can be measured (an operational definition).

This rapid scoping review aimed to identify definitions and attributes of positive psychological capacity to inform the development and validation of a measure of psychological capacity in older people.

The review aimed to explore a breadth of definitions and attributes, from studies of various designs, but not to critique the quality of included papers. We determined that a scoping review would be best suited to our objectives (Munn et al. 2018). A rapid review approach was adopted so that findings could be generated in a timely manner to inform discussion by an expert panel.

*Review question*

- What attributes and definitions of positive psychological capacity or psychological capital exist in the literature?

## Methods/Design

This rapid scoping review was conducted using modified JBI methodology for scoping reviews (Peters et al. 2020), which sets out a systematic and strategic approach to literature search, selection and analysis according to predetermined review questions, parameters and criteria. Guidance from the Cochrane rapid review recommendations (Garritty et al. 2024) also informed the methodology. The review is reported according to PRISMA-ScR guidelines (see Appendix I). Rapid reviews follow systematic and explicit methods, but limit some of the procedures in order to develop evidence in a shorter time frame (Garritty et al. 2024). In this study, knowledge users were not involved, the search was limited to the past 15 years (2010–2025) and the screening and data extraction tools were not piloted. Furthermore, data were extracted by a single reviewer instead of two independent reviewers to reduce the time frame. The objectives, inclusion criteria and methods for this scoping review were registered a priori (https://osf.io/aeqfs).

### Eligibility criteria

#### Participants

Studies relating to human participants were eligible for inclusion. No age limitations were applied to allow broad exploration of the concept.

#### Concept

Eligible studies provided either a specific conceptual or operational definition of psychological capacity or psychological capital, or named attributes as being related to psychological capacity or capital.

#### Context

Eligible studies used a positive psychology or strength-based framework or approach or were otherwise identifiable as being written from a positive psychology or strength-based premise. Studies using the term “psychological capacity” in the context of mental illness or from a deficit viewpoint were excluded.

### Search strategy

A search strategy was developed by the research team using an iterative testing process and was translated across four databases: PsycINFO, PubMed, Embase and Scopus. Keywords used were “psychological capacity” searched with the ‘text word’ field code in PsycINFO, EMBASE, and PubMed, and with title, abstract and keywords in Scopus as these are the field codes closest in scope between these databases (see Appendix II). No subject headings map to these terms, so none were used. Searches were conducted in April 2025 and included English-language publications from 1 January 2010 to 16 April 2025. This search time frame was selected based on a preliminary search that indicated growth in use of this term in the academic literature, particularly beyond the field of organizational psychology, from around 2010 to 2011. Furthermore, the WHO World Report on Ageing (World Health Organization 2015) was published in 2015, incorporating the concept of intrinsic capacity, and we sought to capture the literature that preceded that.

### Study/source of evidence selection

All identified citations from the searches were collated and uploaded into Covidence systematic review software (Veritas Health Innovation, Melbourne, Australia) for deduplication and screening. Titles and abstracts were screened by two reviewers working independently using a liberal approach to inclusion at this stage to enable maximum access to potentially eligible data. Potentially relevant sources were retrieved in full text and were assessed against the criteria by two reviewers independently. Any conflicts were resolved through discussion.

### Data extraction

Data were extracted using a custom data extraction tool in Covidence (Veritas Health Innovation, Melbourne, Australia) designed by the authors using an iterative testing and revision process. Data were extracted from each included study by one reviewer and checked for accuracy by another. Extracted data included citation details, definitions of psychological capacity and whether they were conceptual (what the concept means) or operational (how to measure it), source of the definition, scope/context of the definition, the population the definition was used in relation to, attributes of psychological capacity, any associated theory and theory author (if supplied).

### Data analysis and synthesis of results

The extracted data were exported into MS Excel. Characteristics of included studies were collated into tables and figures and described in a narrative summary. Psychological capacity attributes were inductively identified from the data (Appendix III includes the list of identified attributes, prior to grouping). These attributes were entered into MS CoPilot AI (Basic, with Microsoft 365), which was asked to group them into categories. AI was used solely to perform the initial grouping of attributes previously identified by the research team and did not generate new attributes or concepts. The research team carefully checked, discussed and refined the groupings without further AI use, providing the human oversight recommended by the RAISE guidelines for AI use in evidence synthesis (Flemyng et al. 2025). Each attribute was classified as ‘experience/outcome’, ‘process’ or ‘antecedent’ by consensus among the research team. Definitions and descriptions of psychological capacity and psychological capital were also extracted and collated.

## Results

**Fig. 1:**
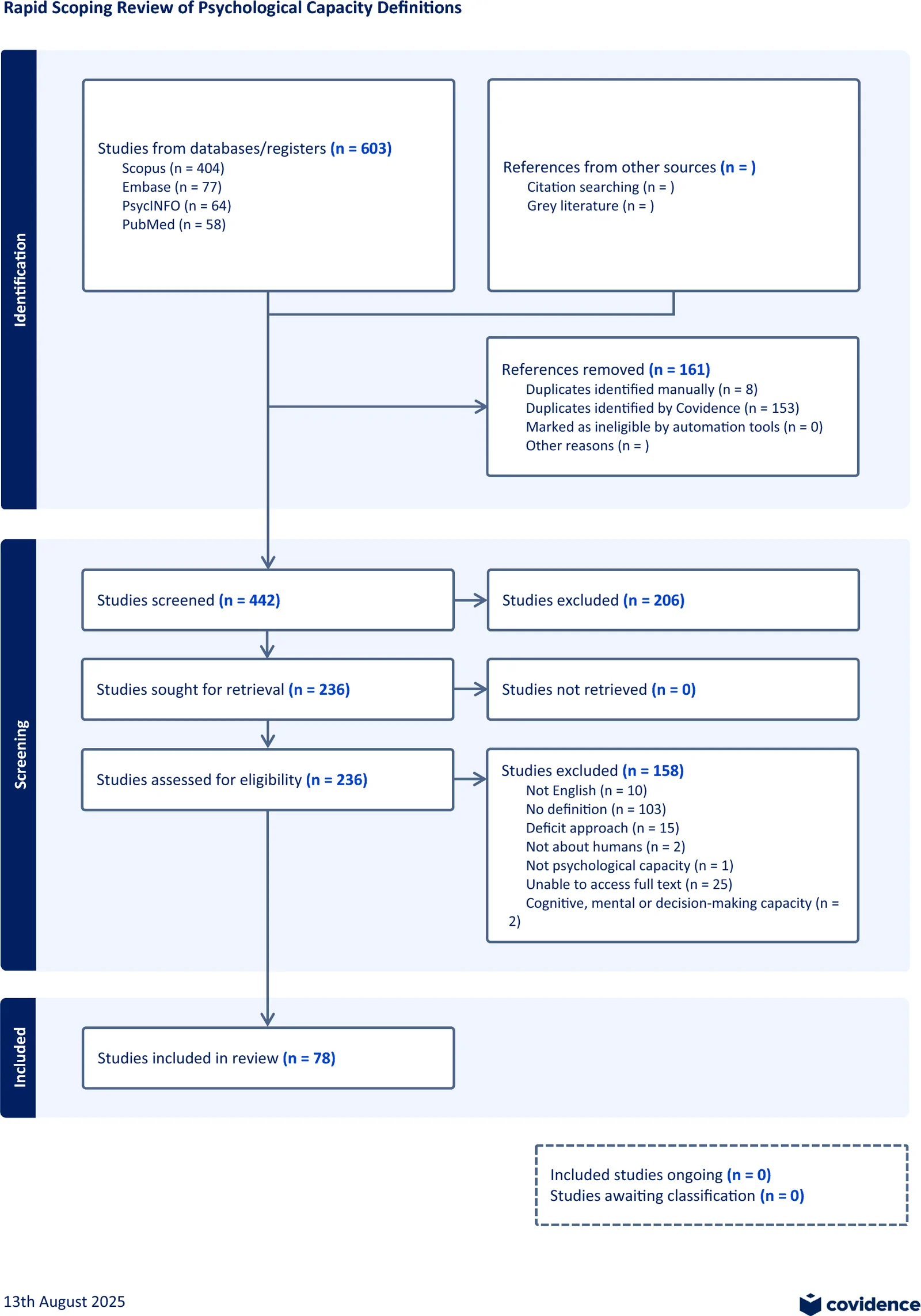
Rapid scoping review of psychological capacity definitions

Of the 603 records retrieved from searches, 78 papers met the criteria and were included. The PRISMA diagram (Fig. 1) shows the study selection process and reasons for full-text exclusion. Details of included studies are in Appendix IV, and excluded studies are in Appendix V.

### Characteristics of included studies

The majority of studies (*n* = 46, 59%) related to the workplace context, while 26 (33%) did not specify. Six studies related to other contexts (see Table 1). Most studies (*n* = 53, 68%) did not refer to a theory. Twenty-one (27%) referred to positive psychology and one each referred to ‘Levels of Emotional Awareness’, ‘Positive Organizational Behaviour’ and ‘Authentic leadership’ as the underlying theory.

**Table 1:** Distribution of scope of included studies (and associated sources of cited definitions)

Study scope	Number of studies	%	Cited source of definition
Work (incl leadership)	46	59	Most frequent sources are key papers by Luthans et al. (2004, 2007a) and Linden et al. (2018)
Not specified	26	33	N/A
Health behaviour	1	1.2	Not specified
Health (surgery)	1	1.2	Luthans (2003)
Academic adjustment	1	1.2	Luthans (2006)
Education	1	1.2	Luthans (2006)
Mental health	1	1.2	Not specified
Parenting	1	1.2	Not specified

The majority of papers (*n* = 61, 78%) did not specify an age range and simply described their sample as an "adult population". Only two (2.5%) related to older adults aged over 65. Table 2 shows the populations reported in the included studies.

**Table 2:** Distribution of participant types across included studies

Population	Number of papers	%
Adults (incl workers)	61	78
None given	8	10
People with chronic diseases	3	4
Older adults (> 65)	2	2.5
Students	2	2.5
Sample Aged 14–95	1	1.2
Youth	1	1.2

### Identified definitions of psychological capacity

We identified eight definitions of psychological capacity in the included studies (Table 3).

**Table 3:** Definitions of psychological capacity given in included studies

Definition	Source	# included studies in which it was cited
“… an individual's positive psychological state of development and is characterised by: (1) having confidence (self-efficacy) to take on and put in the necessary effort to succeed at challenging tasks; (2) making a positive attribution (optimism) about succeeding now and in the future; (3) persevering towards goals and, when necessary, redirecting paths to goals (hope) in order to succeed; and (4) when beset by problems and adversity, sustaining and bouncing back and even beyond (resilience) to attain success.”[31 p1]	Luthans et al. (2004, 2007a, 2006, 2007b, 2016; 2003; 2002a, 2002b; 2004, 2012; 2017) plus several other related sources	*n* = 38, 49%
“Psychological capacities can be described as specific psychological activities which an individual is able to carry out, given specific context requirements”	Linden et al. (2018; 2009)	n = 4, 5%
“… (1) the maximum ability of an individual to receive or retain information and knowledge or to function in mental or physical tasks; (2) the potential of an individual for intellectual or creative development or accomplishment; (3) inborn potential, as contrasted with developed potential (see ability)”	APA Dictionary of Psychology (2018)	n = 1, 1% Gill et al. (2024)
“… the term ‘psychological capacity' refers to an individual's ability to use self-motivated behavior to take on and overcome challenges to achieve positive outcomes in life or work”	Brady et al. (2019) p393	n = 1, 1% Ewe et al. 2022 ( 2022)
"… the intrinsic characteristics that motivate working adults to persist in workplace excellence."	Luthans et al. (2006) p209	n = 1, 1% Black (2023)
“… the capacity to perceive and understand the world, to form projects, to reflect and deliberate on these projects, and so on. Chief among these capacities is autonomy"	Not specified	n = 1, 1% Kerstein (2011)
"Psychological capacities refer to development of psychological potential savings, and the ability to withstand the outside pressure, usually including psychological potential, anti-frustration ability and ability to overcome adversity."	Not specified	n = 1, 1% Li et al. (2013)
“The mental and emotional adjustment to achieving self-management and psychological resilience; …”	Not specified	n = 1, 1% Young-Silva (2024)

Various types of definition for psychological capacity were reported. Seventy papers (90%) provided a descriptive definition or elaboration. An ‘operational’ definition was provided in 41 papers (53%), and a ‘conceptual’ definition in 39 papers (50%). Twenty-seven papers (35%) included both types of definition as well as attributes of psychological capacity.

With regard to terminology, twenty-two (28%) papers used both 'psychological capacity’ and ‘psychological capital’, 35 (45%) used only ‘psychological capacity/capacities’ and 12 (15%) used only ‘psychological capital’ and/or ‘PsyCap’.

Some studies also included descriptions of psychological capacity/capital and the traits thereof. Several studies cited Luthans’ (Luthans et al. 2004; Luthans et al. 2007a; Luthans et al. 2006; Luthans et al. 2007b; Luthans 2002b; Luthans and Youssef 2004) explanations of psychological capital as being “… state-like and open to development (as opposed to trait-like and relatively fixed)” (Luthans and Youssef 2012; Luthans et al. 2016; Nolzen 2018; Koutsoumari and Antoniou 2016; Baykal and Zehir 2018; Goertzen and Whitaker 2015; Toprak et al. 2024). A more detailed explanation was also given as:

*“PsyCap traits are positioned in the middle of a continuum as they are more stable and less changeable than emotions and moods, which are momentary, changeable, and unstable. PsyCap traits are also more trainable and open to development compared to other traits like personality, which are more rigid and fixed, especially in adults. In other words, based on criteria of stability and trainability, emotions fall on the left of the continuum as they are more short-lived and highly transient, PsyCap traits fall in the middle of the continuum as they are more flexible and trainable traits. In contrast, personality traits are located on the right of the continuum because they are less flexible and more hard-wired”* (Luthans (Luthans and Youssef-Morgan 2017), cited in Toprak 2024 [52 p630-1]).

DeWitt 2018 (DeWitt et al. 2018) referred to the distinction between ‘self capacities’ (e.g. hope, regulation, persistence etc.) and ‘relationship capacities’ (e.g. empathy, reciprocity etc.). In three studies, psychological capacity was likened to ‘soft skills’ (Henning et al. 2023; Muschalla 2024, 2025) “which are essential in today’s working life with increased psychological requirements and which enable individuals to interact with others and to adjust to different situations or requirements” [54 p587].

### Key attributes of psychological capacity

Fifty-six attributes of psychological capacity were mentioned in the included studies. The most frequently occurring were the four attributes included in Luthans et al.’s (Luthans et al. 2006) definition of psychological capacity: Hope, Self-efficacy, Resilience and Optimism. Table 4 lists the attributes, their frequency and the papers in which they were identified. It also shows indicative alignment with whether the attributes constitute antecedents, processes or outcomes of psychological capacity. The attributes were grouped into nine domains: psychological; resilience and adaptability; cognitive process/function; emotion; social and interpersonal competence; self-identity, values, and agency; physical and spatial–temporal orientation; behaviour; and well-being and life satisfaction. Attributes from the ‘psychological’ and ‘resilience and adaptability’ domains were most frequently identified. Thirty-four attributes were classified as ‘Experience/Outcome’ attributes (*n* = 34), thirty were classified as ‘Process’ (state-like) attributes, and 14 were classified as ‘Antecedent’ (trait-like) attributes. There was also overlap, with 15 attributes being classified as both ‘Process’ and ‘Experience/Outcome’, and four classified as both ‘Antecedent’ and ‘Process’.

**Table 4:** Key attributes of psychological capacity

Attributes	Cited source	Included papers which listed this attribute	Frequency in included studies	Antecedent (Trait-like)	Process (State-like)	Experience (Positive outcome)
*Psychological*			55			
Self-efficacy (or confidence)	Luthans and Avolio (2003), Luthans et al. (2006)	Luthans and Youssef (2004), Luthans and Youssef-Morgan (2017), Brady et al. (2019), Ewe and Ho (2022), Young-Silva et al. (2024), Koutsoumari and Antoniou (2016), Baykal and Zehir (2018), Muschalla (2025), Alavi and Gill (2017), Avey et al. (2011), Azab et al. (2018), Brunetto et al. (2024a), Brunetto et al. (2024b), Buchko 2022), Burns et al. (2017), Diedericks et al. (2019), Higgins et al. (2010), Huo et al. (2022), Hystad and Eid (2016), Kalay et al. (2020), Kanengoni et al. (2018), Kaur and Ren (2023), Khatri and Dutta (2022), Li (2018), Wu and Kang (2025), Gupta and Singh (2014), Hazan Liran and Miller (2019), Kalman and Summak (2017), Magnano et al. (2016), Mitchell (2016), Newstead (2021)	34			
Hope	Luthans and Avolio (2003), Luthans et al. (2006), Singh (2024)	Oster et al. (2023a), Luthans and Youssef (2004), Luthans and Youssef-Morgan (2017), Brady et al. (2019), Ewe and Ho (2022), Young-Silva et al. (2024), Koutsoumari and Antoniou (2016), Baykal and Zehir (2018), Goertzen and Whitaker (2015), Toprak et al. (2024), Muschalla (2025), Alavi and Gill (2017), Avey et al. (2011), Azab et al. (2018), Brunetto et al. (2024a), Brunetto et al. (2024b), Buchko (2022), Burns et al. (2017), Diedericks et al. (2019), Higgins et al. (2010), Huo et al. (2022), Hystad and Eid (2016), Kalay et al. (2020), Kanengoni et al. (2018), Kaur and Ren (2023), Khatri and Dutta (2022), Li (2018), Wu and Kang (2025), Gupta and Singh (2014), Hazan Liran and Miller (2019), Kalman and Summak (2017), Magnano et al. (2016), Mitchell (2016), Newstead (2021), Zilberg et al. (1991), Alenezi et al. (2024), Almutairi et al. (2025), Chaudhary (2022), Silva Zonatto et al. (2023), Dawkins et al. (2013), Donaldson and Villalobos (2024), English and Dikun (2015), Joaquim et al. (2018), Kavgacı and Öztürk (2023), Liyanagamage et al. (2024), Machado et al. (2022), Sulastiana et al. (2019), Wu and Kang (2023)	48			
Optimism	Luthans and Avolio (2003), Luthans et al. (2006)	Oster et al. (2023a), Luthans and Youssef (2004), Luthans and Youssef-Morgan (2017), Brady et al. (2019), Ewe and Ho (2022), Young-Silva et al. (2024), Koutsoumari and Antoniou (2016), Baykal and Zehir (2018), Goertzen and Whitaker (2015), Muschalla (2025), Alavi and Gill (2017), Avey et al. (2011), Azab et al. (2018), Brunetto et al.( 2024a), Brunetto et al. (2024b), Buchko (2022), Burns et al. (2017), Diedericks et al. (2019), Higgins et al. (2010), Huo et al. (2022), Hystad and Eid (2016), Kalay et al. (2020), Kanengoni et al. (2018), Kaur and Ren (2023), Khatri and Dutta (2022), Li (2018), Wu and Kang (2025), Gupta and Singh (2014), Hazan Liran and Miller (2019), Kalman and Summak (2017), Magnano et al. (2016), Mitchell (2016), Newstead (2021), Zilberg et al. (1991), Alenezi et al. (2024), Almutairi et al. (2025), Chaudhary (2022), Silva Zonatto et al. (2023), Dawkins et al. (2013), Donaldson and Villalobos (2024), English and Dikun (2015), Joaquim et al. (2018), Kavgacı and Öztürk (2023), Liyanagamage et al. (2024), Machado et al. (2022), Sulastiana et al. (2019), Wu and Kang (2023), Xu and Chen (2017)	48			
Perseverance, persistence	Luthans et al. (2004), Flemyng et al. (2025), Luthans and Avolio (2003), Luthans (2002b), Luthans and Youssef (2012), Alavi and Gill (2017), Singh (2024)	Luthans and Youssef-Morgan (2017), Ewe and Ho (2022), Baykal and Zehir (2018), Toprak et al. (2024), Henning et al. (2023), Muschalla (2024), Alavi and Gill (2017), Azab et al. (2018), Burns et al. (2017), Huo et al. (2022), Kanengoni et al. (2018), Wu and Kang 2025), Hazan Liran and Miller 2019), Mitchell 2016), Xu and Chen 2017), Vella et al. (2011)	16			
Proactivity, Motivation	Luthans et al. (2004), Luthans et al. (2007a), Luthans and Avolio 2003)	Henning et al. (2023), Muschalla 2024), Kalman and Summak 2017), Liyanagamage et al. (2024), Machado et al. (2022), Bošnjak 2023), Muschalla and Kutzner 2021)	7			
Attachment to life), outlook on life	Flemyng et al. (2025)	Oster et al. (2023a), Kerstein (2011)	2			
Zest for life	Singh 2024)	Toprak et al. (2024)	1			
Flow experience		Wu and Kang (2023)	1			
*Resilience and Adaptability*			60			
Resilience; anti-setback ability; anti-frustration ability, ability to overcome adversity/challenges	Luthans and Avolio 2003), Luthans et al. (2006)	Oster et al. (2023a), Luthans and Youssef (2004), Luthans and Youssef-Morgan (2017), Brady et al. (2019), Ewe and Ho (2022), Kerstein (2011), Li and Sun (2013), Young-Silva et al. (2024), Koutsoumari and Antoniou (2016), Baykal and Zehir 2018), Goertzen and Whitaker (2015), Muschalla (2025), Alavi and Gill (2017), Avey et al. (2011), Azab et al. (2018), Brunetto et al. (2024a), Brunetto et al. (2024b), Buchko (2022), Burns et al. (2017), Diedericks et al. (2019), Huo et al. (2022), Hystad and Eid (2016), Kalay et al. (2020), Kanengoni et al. (2018), Khatri and Dutta (2022), Li (2018), Wu and Kang (2025), Gupta and Singh (2014), Hazan Liran and Miller (2019), Kalman and Summak (2017), Magnano et al. (2016), Mitchell (2016), Newstead (2021), Zilberg et al. (1991), Alenezi et al. (2024), Almutairi et al. (2025), Chaudhary (2022), Silva Zonatto et al. (2023), Dawkins et al. (2013), Donaldson and Villalobos (2024), English and Dikun (2015), Joaquim et al. (2018), Kavgacı and Öztürk (2023), Liyanagamage et al. (2024), Machado et al. (2022), Sulastiana et al. (2019), Wu and Kang (2023), Xu and Chen (2017), Muschalla (2022), Abro et al. (2023), Fladerer and Braun (2020)	51			
Flexibility, Immediate adaptation	Luthans et al. (2007a), Luthans and Avolio (2003), Luthans et al. (2016), Singh (2024)	Toprak et al. (2024), DeWitt et al. (2018), Henning et al. (2023), Muschalla (2024), Brunetto et al. (2024a), Bošnjak (2023), Muschalla and Kutzner (2021), Joker et al. (2022), Frey et al. (2025), Muschalla (2018), Neue and Muschalla (2024)	11			
Endurance (psychological)	Luthans et al. (2016), Luthans et al. (2007a)	Kerstein (2011), DeWitt et al. (2018), Henning et al. (2023), Muschalla (2024), Bošnjak (2023), Muschalla and Kutzner (2021), Frey et al. (2025), Muschalla (2018)	8			
Psychological potential		Kerstein (2011)	1			
*Cognitive process/function*		17				
Decision making and judgement	Luthans et al. (2016)	DeWitt et al. (2018), Henning et al. (2023), Muschalla (2024), Bošnjak (2023), Muschalla and Kutzner (2021), Frey et al. (2025), Muschalla (2018), Nevejan (2024)	8			
Structuring and planning tasks; capacity to form projects, reflect and deliberate	Luthans et al. (2016)	Black et al. (2023), DeWitt et al. (2018), Henning et al. (2023), Muschalla (2024), Muschalla and Kutzner (2021), Frey et al. (2025), Muschalla (2018)	7			
Perception, capacity to perceive and understand the world, General self/world comprehension	Nusbaum (2025)	Black et al. (2023), Vella et al. (2011), Graham (2013)	3			
Apply knowledge, apply expertise, competency	Luthans et al. (2007a)	Henning et al. (2023), Bošnjak (2023), Muschalla and Kutzner (2021)	3			
Cognitive resources/functioning	Luthans et al. (2004)	Hazan Liran and Miller (2019), Kalman and Summak (2017), Machado et al. (2022)	3			
Memory, retain information and knowledge	Linden et al. (2009)	American Psychological Association et al. (2018), Graham 2013)	2			
Attention		Graham 2013)	1			
Receive information	Linden et al. (2009)	American Psychological Association et al. (2018)	1			
Language		Graham (2013)	1			
Rational thinking		Nevejan (2024)	1			
Recognition of opportunities or affordances	Nusbaum (2025)	Vella et al. (2011)	1			
Imagination		Neue and Muschalla (2024)	1			
*Emotion*		15				
Regulation (affect, emotion/al, ‘self’, impulse, sexual)	Singh (2024)	Oster et al. (2023a), Toprak et al. (2024), Graham (2013), Love (2024), Bram and Peebles (2014), Kempe et al. (2024), Kok et al. (2013)	7			
Emotional intelligence; affect processing	Luthans et al. (2006)	Higgins et al. (2010), Kaur and Ren (2023), Machado et al. (2022), Nevejan (2024), Volkert et al. (2021)	5			
Mindfulness		Oster et al. (2023a)	1			
Patience	Gill and Singh (2024)	Brady et al. (2019)	1			
Insight into one’s developmental histories and psychological vulnerabilities		Subic-Wrana et al. (2011)	1			
Forgiveness		Xu and Chen (2017)	1			
*Social and Interpersonal Competence*		13				
Assertiveness; assertion	Luthans et al. (2016), Singh (2024)	Toprak et al. (2024), DeWitt et al. (2018), Henning et al. (2023), Muschalla (2024), Bošnjak (2023), Muschalla and Kutzner (2021), Frey et al. (2025), Muschalla (2018)	8			
Group integration; group and teamwork capacity	Luthans et al. (2007a), Luthans et al. (2016)	DeWitt et al. (2018), Henning et al. (2023), Muschalla (2024), Bošnjak (2023), Frey et al. (2025), Muschalla (2018)	6			
Dyadic (familiar/intimate) relationships; contact with others	Luthans et al. (2007a)	Henning et al. (2023), Muschalla (2024), Bošnjak (2023), Muschalla and Kutzner (2021), DeWitt et al. (2018), Muschalla (2018)	6			
Social interaction; social skills; social cognition		Henning et al. (2023), Muschalla (2024), Machado et al. (2022), Graham (2013)	4			
Commitment	Singh (2024)	Toprak et al. (2024), Vella et al. (2011), Fladerer and Braun (2020)	3			
Empathy; mentalizing	Singh (2024)	Toprak et al. (2024), Kok et al. (2013)	2			
Communication	Nusbaum (2025)	Vella et al. (2011)	1			
Consideration		Brady et al. (2019)	1			
Reciprocity	Singh (2024)	Toprak et al. (2024)	1			
Trust	Singh (2024)	Toprak et al. (2024)	1			
Reliance	Singh (2024)	Toprak et al. (2024)	1			
*Self-Identity, Values, and Agency*			7			
Self-coherence, Sense of coherence	Singh (2024)	Oster et al. (2023a), Toprak et al. (2024), Bram and Peebles (2014)	3			
Responsibility; attribution of responsibility; responsibility for self	Singh (2024), Nusbaum (2025)	Brady et al. (2019), Toprak et al. (2024), Vella et al. (2011)	3			
Self-esteem	Singh (2024)	Toprak et al. (2024)	1			
Standards and values	Singh (2024)	Toprak et al. (2024)	1			
Autonomy	Black et al. (2023)		1			
Personality functioning	Kok et al. (2013)		1			
*Physical and Spatial–Temporal Orientation*		7				
Mobility	Luthans et al. (2016)	DeWitt et al. (2018), Henning et al. (2023), Muschalla (2024), Muschalla and Kutzner (2021), Frey et al. (2025), Muschalla 2018)	6			
Physical fitness	Luthans et al. (2016)	Frey et al. (2025)	1			
Bodily/spatial self-location	Nusbaum (2025)	Vella et al. (2011)	1			
Historical/temporal self-location	Nusbaum (2025)	Vella et al. (2011)	1			
*Behaviour*			6			
Adherence to regulations/rules	Luthans et al. (2016)	DeWitt et al. (2018), Henning et al. (2023), Muschalla (2024), Muschalla and Kutzner (2021), Frey et al. (2025), Muschalla 2018)	6			
Self-care; care for oneself	Luthans et al. (2007a)	DeWitt et al. (2018), Henning et al. (2023), Muschalla (2024), Bošnjak (2023), Muschalla and Kutzner (2021), Muschalla (2018)	6			
*Well-Being and Life Satisfaction*			2			
Evaluative (subjective) well-being	Luthans et al. (2006)	Kaur and Ren (2023), Helle et al. (2023)	2			
Eudemonic well-being		Helle et al. (2023)	1			
Hedonic well-being		Helle et al. (2023)	1			

## Discussion

The concept of psychological capacity has gained increasing attention in recent years, yet its meaning and scope have not been clearly articulated. To address this gap, this rapid review is the first to systematically map how psychological capacity has been defined and operationalized in the international literature. While the concept of psychological capacity is articulated through multiple definitions and descriptions, our review has found that no specific definition of psychological capacity for healthy ageing exists.

We identified eight definitions, three related descriptions and 56 attributes of psychological capacity (grouped into nine domains) across the 78 included papers. Hope, self-efficacy, resilience and optimism were the most frequently identified attributes, drawing on Luthans’ concept of psychological capital (Luthans et al. 2004). Attributes were predominantly classified as ‘Experience/Outcome’ attributes (*n* = 34), or ‘Process’ (state-like) attributes (*n* = 30), and 14 were classified as ‘Antecedent’ (trait-like) attributes, and there was considerable overlap. These findings illustrate that psychological capacity is a complex, multidimensional concept encompassing the mental, emotional, and social resources that enable individuals to adapt, function, and thrive across life contexts.

The dominant framework and body of literature by Luthans and colleagues that underlies most research in this area is drawn from the ‘work’ setting. This is oriented towards goals of ‘positive organizational behaviour’ in ‘human *resources*’ that can be “developed and managed for *performance impact*” [117 p20] and competitive advantage [emphasis added]. The advantage of this foundational psychological capital/capacity work for the concept of psychological capacity is that it was motivated by adopting positive psychology ideas, pursuing strength-based approaches, and moving away from deficit focussed conceptions. However, healthy ageing is not concerned with performance and competitive advantage, rather it focusses on intrinsic capacity (for all people) to live well in older age. As such, the definition and attributes of psychological capital/capacity stemming from Luthans’ work cannot necessarily be directly applied to psychological capacity for healthy ageing.

Nonetheless, this work provides a useful starting point to develop and test conceptual and operational definitions for psychological capacity in the context of healthy ageing that aligns with the ethos of intrinsic capacity. Luthans and colleagues’ higher-order construct of psychological capital was defined as a state-like variable that is positive, unique, measurable and able to be developed. They included four attributes that fit with these criteria (self-efficacy, hope, optimism and resilience) in the higher-order construct of psychological capital. While these four attributes have been studied in the context of older people, there is limited understanding of a higher-order construct of psychological capacity for healthy ageing. It is also not clear what would constitute the attributes that when combined as a ‘whole’ would be greater than individual attributes (or the sum of its parts) in healthy ageing.

There was very little to indicate that the definitions identified in the included studies drew upon other theories. However, in related work, Luthans et al. (2007c) and Youssef-Morgan et al. (2013) explain that their concept of PsyCap (psychological capital) is founded on other theories such as Bandura’s Social Cognitive Theory (Bandura 1986); Snyder’s Hope theory (Snyder 2000) and Fredrickson’s ‘broaden-and-build’ model (Fredrickson 2004).

It is worth noting that some of the attributes we have identified in the included studies potentially overlap or are better aligned with other WHO domains of intrinsic capacity (World Health Organization 2015). For example, twelve of the attributes identified in this review have been grouped together as ‘Cognitive process/function’ attributes (including ‘decision making and judgement’, ‘structuring and planning tasks’, and ‘perception’, to list some of the more frequent ones). Such mental capacities arguably more closely align with the ‘Cognition’ domain of intrinsic capacity. Furthermore, some of the attributes we have identified and grouped as ‘Physical and spatial–temporal orientation’ (e.g. mobility, physical fitness) arguably align better with the ‘Locomotion’ domain of intrinsic capacity. Further refinement to confirm the attributes of psychological capacity for healthy ageing is required and is planned to follow this initial review.

While our study has identified a comprehensive range of attributes, there is limited understanding of how the attributes interact and disassociate antecedence, process and outcome/experience. Furthermore, there is a need to understand the causal mechanisms that produce them. For example, what causes someone to have resilience, or what causes someone to have hope? Such knowledge is essential to inform the design of effective individual and population interventions to promote and enhance psychological capacity for healthy ageing, across the life course, and spanning community and residential aged care settings.

### Limitations

Key methodological limitations of this review stem from the fact that it was rapid review that did not extend to the full methodology of a scoping review. The review did not involve or engage with knowledge users. The search was limited to English-language published articles, which may have excluded relevant grey literature or studies published in other languages. The search time frame was also limited to the past 15 years.

The screening criteria and data extraction tools were not piloted prior to conducting these elements of the research (as is recommended for a scoping review); however, these sections of the methods were carried out successfully, and included studies were screened and analyzed appropriately to achieve the review aim. Data extraction could have been strengthened by having data independently extracted by two reviewers; however, our approach of single data extraction with checking by a second reviewer was sufficiently robust and allowed a large range of attributes to be identified. No formal quality appraisal of included studies was undertaken, consistent with scoping and rapid review methodology, which may limit the interpretive depth of the findings.

### Future directions

Despite these limitations, the rapid review results provide a useful starting point for developing and validating a measure of psychological capacity for healthy ageing. The identified attributes of psychological capacity that we have identified in this review will serve as a starting point for establishing conceptual and operational definitions of psychological capacity for healthy ageing, through a process of expert consensus. An International Working Group of experts will develop a draft definition based on the identified attributes. From there, a three-round Delphi survey process will gain expert consensus to finalize the definition and attributes. The outcome of the Delphi process will be an operational definition of psychological capacity, and a list of items for a scale to measure psychological capacity, which will subsequently undergo translation and cultural adaptation, psychometric testing, and validation and reliability testing.

## Conclusions

The World Health Organization’s (WHO) has proposed ‘intrinsic capacity’ as a key determinant of healthy ageing. Psychological capacity is one of the WHO’s five domains of capacity, but lacks definitional clarity, hampering efforts to measure progress towards healthy ageing. This rapid review provides an important starting point towards developing and validating a measure of psychological capacity for health ageing. Most studies were conducted in workplace and organizational settings, highlighting a gap in research applying these attributes to healthy ageing and everyday functioning in later life. This indicates a need to adapt and validate the concept of psychological capacity within the WHO’s intrinsic capacity framework to ensure that it reflects the realities of ageing populations across diverse cultural contexts. Multiple definitions and attributes of psychological capacity were identified in this rapid review, and these were grouped into preliminary ‘domains’. The findings from this review provide a robust foundation of evidence to inform the development of a valid, applicable and culturally appropriate measure of psychological capacity for global use.

## Supplementary Information

Below is the link to the electronic supplementary material.

Supplementary file1 (PDF 645 kb)

Supplementary file2 (DOCX 21 kb)

Supplementary file3 (DOCX 27 kb)

Supplementary file4 (CSV 17 kb)

Supplementary file5 (CSV 41 kb)

## Data Availability

No datasets were generated or analyzed during the current study.
