# Supplementary material for: Defining psychological capacity for a measure of healthy ageing: a rapid scoping review
Source: Eur J Ageing. 2026 Jul 11;23(1):38. doi: 10.1007/s10433-026-00933-w (PMC13642232; doi:10.1007/s10433-026-00933-w)
Supplement: Supplementary file 2 [file 10433_2026_933_MOESM2_ESM.docx]

Appendix II – Search terms and results

| Data base | Search terms | Studies retrieved |
| --- | --- | --- |
| PsycInfo | "psychological capacity".tw. Limit 2010-2025 | 64 |
| Embase | "psychological capacity".tw. Limit 2010-2025 | 77 |
| Scopus | TITLE-ABS-KEY "psychological capacity" | 404 |
| Pubmed | "psychological capacity"[Text Word] Limit 2010-2025 | 58 |
