# Supplementary material for: Defining psychological capacity for a measure of healthy ageing: a rapid scoping review
Source: Eur J Ageing. 2026 Jul 11;23(1):38. doi: 10.1007/s10433-026-00933-w (PMC13642232; doi:10.1007/s10433-026-00933-w)
Supplement: Supplementary file 3 [file 10433_2026_933_MOESM3_ESM.docx]

**Appendix III**

**Identified attributes (un-grouped)**

**Attribute**

Adherence to regulations/rules

Affect processing

Affect regulation; emotion/al regulation

Anti-frustration ability

Anti-setback ability, ability to overcome adversity/challenges

Apply knowledge, apply expertise

Assertion (a relationship capacity)

Assertiveness

Attachment to life

Attention

Attribution *(not otherwise specified)*

autonomy

Bodily/spatial self location

Capacity to form projects, to reflect and deliberate on these projects

Care, commitment and emotional engagement

Cognitive (resources, functioning)

Commitment (a relationship capacity), professional commitment

Communication

Consideration

Contact with others

Decision making and judgement (judgment), capacity to judge and decide,

Dyadic (familiar and intimate) relationships

Emotion

Emotion/al regulation

Emotional engagement, care

Emotional intelligence

Empathy (a relationship capacity)

Endurance (psychological)

Eudemonic wellbeing

Evaluative wellbeing

Expertise and competency, applying expertise

Flexibility

Flexibility in biorhythm

Flow experience

Forgiveness

Function in mental or physical tasks

General self/world comprehension

Group integration, group and team work capacity

Hedonic wellbeing

Historical/temporal self-location

Hope

Imagination

Immediate adaptation

Impulse regulation

Insight into their own developmental histories and resulting psychological vulnerabilities

Language

Memory

Mentalizing

Mindfulness

Mobility

Motivation

Optimism

Outlook on life

Patience

Perception, capacity to perceive and understand the world

Perseverance

Persistence

Personality functioning

Physical fitness

Proactivity

Psychological fitness (concentration, endurance, cognitive and behavioural flexibility)

Psychological potential

Rational thinking

Receive information

Reciprocity (a relationship capacity)

Recognition of opportunities or affordances

Reliance (a relationship capacity)

Resilience

Responsibility for self

Responsibility, attribution of responsibility

Retain information and knowledge

Self-efficacy (confidence)

Self esteem

Self-care, care for oneself

Self-coherence

Self-regulation

Sense of coherence

Sexual regulation

Social cognition

Social interaction, social skills like contacting others

Standards and values

Structuring and planning tasks

Subjective wellbeing

Trust (a relationship capacity)

Zest for life
